# Pilot Field Trial of the EG95 Vaccine Against Ovine Cystic Echinococcosis in Rio Negro, Argentina: Second Study of Impact

**DOI:** 10.1371/journal.pntd.0004134

**Published:** 2015-10-30

**Authors:** Edmundo Larrieu, Guillermo Mujica, Charles G. Gauci, Katherina Vizcaychipi, Marcos Seleiman, Eduardo Herrero, José Luis Labanchi, Daniel Araya, Luis Sepúlveda, Claudia Grizmado, Arnoldo Calabro, Gabriel Talmon, Thelma Verónica Poggio, Pablo Crowley, Graciela Cespedes, Graciela Santillán, Mariela García Cachau, Roberto Lamberti, Lilia Gino, Meritxell Donadeu, Marshall W. Lightowlers

**Affiliations:** 1 Facultad de Ciencias Veterinarias, Universidad Nacional de La Pampa, General Pico, Argentina; 2 Escuela de Veterinaria, Universidad Nacional de Río Negro, Choele Choel, Argentina; 3 Ministerio de Salud, Provincia de Río Negro, Viedma, Argentina; 4 Faculty of Veterinary and Agricultural Sciences, University of Melbourne, Werribee, Australia; 5 Departamento de Parasitología "INEI- ANLIS", Buenos Aires, Argentina; 6 Centro de Virología Animal (CEVAN), Instituto de Ciencia y Tecnología / Cesar Milstein-CONICET, Buenos Aires, Argentina; Universidad Peruana Cayetano Heredia, PERU

## Abstract

**Background:**

Cystic echinococcosis (CE) is an important zoonotic disease caused by the cestode parasite Echinococcus granulosus. It occurs in many parts of the world where pastoral activities predominate, including the Rio Negro province of Argentina. Although CE control activities have been undertaken in the western regions of Rio Negro for more than two decades, the disease continues to remain prevalent in both the human and livestock animal populations. Vaccination of animal intermediate hosts of CE with the EG95 vaccine may provide a new opportunity to improve the effectiveness of CE control measures, although data are lacking about field application of the vaccine.

**Aims:**

Evaluate the impact of EG95 vaccination in sheep on the transmission of Echinococcus granulosus in a field environment.

**Methodology:**

Two trial sites were established in western Rio Negro province within indigenous communities. Vaccination of lambs born into one trial site was introduced and continued for 6 years. Prior to initiation of the trial, and at the end of the trial, the prevalence of CE in sheep was determined by necropsy. Weaned lambs received two injections of EG95 vaccine, approximately one month apart, and a single booster injection one year later. Vaccination was not implemented at the second trial site. A total of 2725 animals were vaccinated in the first year. Animals from this cohort as well as age-matched sheep from the control area were evaluated by necropsy.

**Key results:**

Introduction of the vaccine led to a statistically significant in the number and size of hydatid cysts in comparison to the situation prior to the introduction of the vaccine, or compared to CE prevalence in the control area where the vaccine was not applied. The prevalence of infection in the vaccinated area was also significantly reduced by 62% compared to the re-intervention level, being lower than the prevalence seen in the control area, although the difference from the control area after the intervention was not significant possibly due to limitations in the numbers of animals available for necropsy.

**Conclusions:**

Vaccination of sheep with the EG95 vaccine provides a valuable new tool which improves the effectiveness of CE control activities. Vaccination was effective even in a difficult, remote environment where only approximately half the lambs born into the communities were fully vaccinated.

## Author Summary

Hydatid disease, otherwise known as cystic echinococcosis (CE), is caused by *Echinococcus granulosus*. The disease is common in many pastoral areas, including parts of the Rio Negro province of Argentina, and is formally recognised by the WHO as a Neglected Tropical Disease. We undertook the first scientific evaluation of the EG95 vaccine against transmission of hydatid disease in a field situation. Sheep in remote indigenous communities of Rio Negro were vaccinated over a six year period. Young lambs received two injections of vaccine and a single booster injection as one year old sheep. A similar region acted as a control where vaccination was not implemented. Evaluation of the outcomes of vaccination revealed a significant, 62% decrease in the prevalence of hydatid infection in 6 year old sheep. Our study provides a template for the application of vaccination in future efforts to control transmission of *E*. *granulosus* and reduce the burden of human disease caused by the parasite.

## Introduction

Cystic echinococcosis (CE) is a zoonosis present worldwide produced by *Echinococcus granulosus* (EG). As the most common intermediate hosts (sheep and goats) that develop cysts in liver and lung [1] [2], human may be infected and develop cysts in the same organs. The dog is the definitive host.

CE appears mostly in rural areas dedicated to the breeding of small ruminants, and it is associated to the practice of feeding dogs with viscera from the slaughter of infected sheep. Most of the 2000 new human cases diagnosed every year in the Americas originate from these areas. However, the migratory movements to peri-urban areas by rural populations that maintain small flocks of sheep and preserve the practice of feeding viscera to their dogs have increased the geographical distribution of the disease and its potential public health impact.

The health burden of CE is highlighted through its recognition by the World Health Organization as a Neglected Tropical Disease [3].

In Rio Negro Province in Argentine, the prevalence of the infection by *Echinococcus granulosus* in dogs, sheep and human was very high causing a significant burden for the health system due to the high costs of surgery and days spent in hospital [4,5]. In 1980, the incidence was 146 (38 x 100000). In this year started a control program, based on systematic deworming of dogs with praziquantel (health care assistants of existing primary health care visited rural areas and distributed praziquantel tablets to dog owners who were ultimately responsible for carrying out the deworming), slaughter control, health education and included strategies to improve patient prognosis through early detection and timely treatment, especially in the young population [6–8].

The experience in Rio Negro province has been successful in obtaining important decreases in transmission to human and dogs. These efforts, may not have attained the planned deworming coverage or an important effect on sheep, and this has been sufficient to lead to a continued incidence of CE in children and adults [9–11].

Vaccination of potential intermediate hosts of EG with the EG95 recombinant vaccine [12,13] could potentially be used to reduce the level of EG transmission and decrease the incidence of human infections, which it was also estimated based on mathematical models, including the association with praziquantel in definitive hosts [14–16].

The control program in Río Negro decided the introduction of the vaccine as an additional control tool in some areas of the province. Vaccination program began in December 2009 and a preliminary evaluation of the impact was undertaken using serological methods in 2012 [10] and showed a significant reduction in CE was evident in the vaccinated animals (p<0.01). This is an unique study about the impact of the EG95 vaccine used in field conditions and potential problems that could arise when the vaccine is applied on a large scale in livestock.

The objective of this work was to undertake a more thorough assessment of the effects of EG95 vaccination on lambs born following the introduction of the vaccine

## Materials and Methods

**Fig 1:**
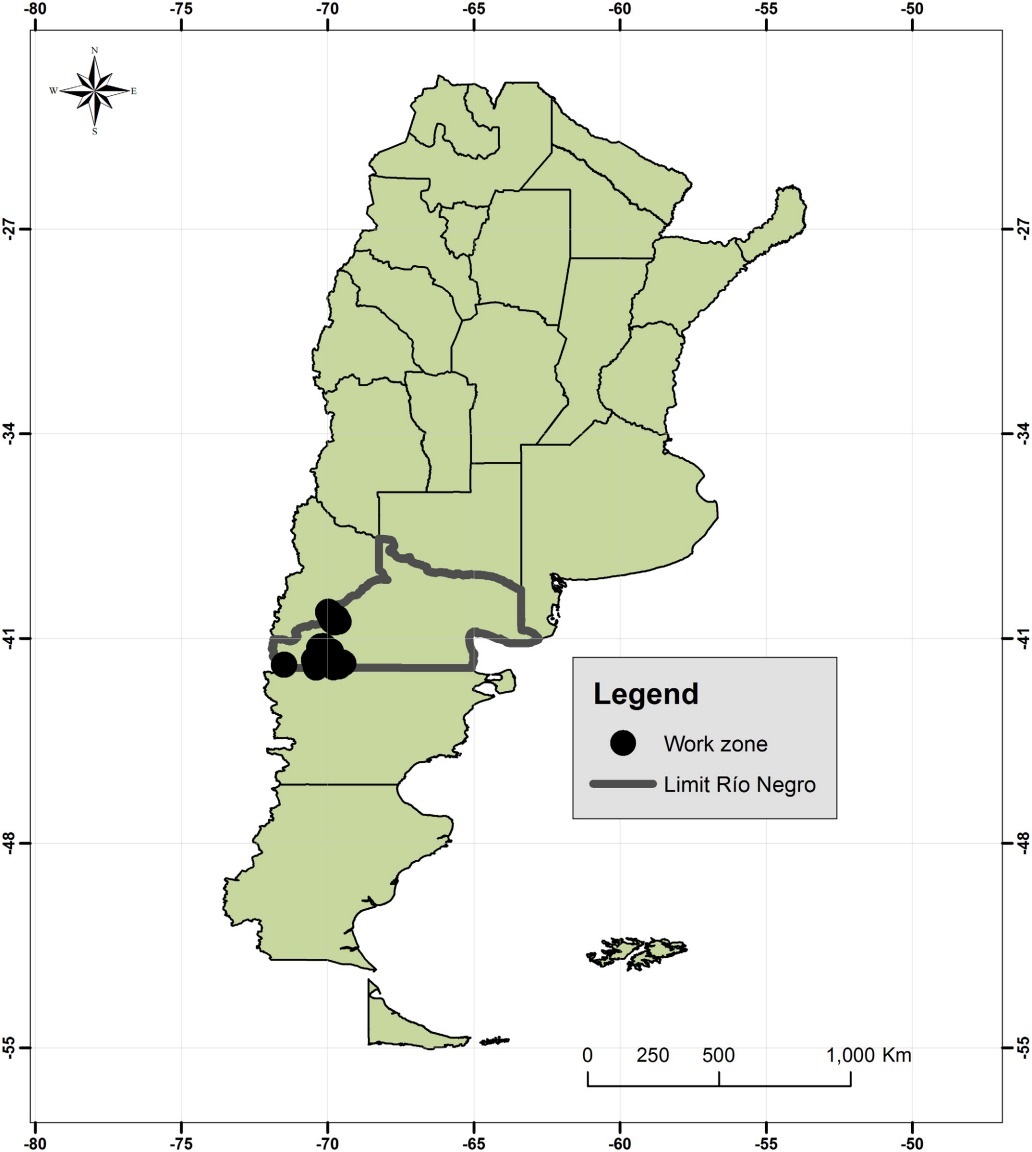
Geographic area for CE control programme showing sheep farm in control and vaccination areas. Río Negro, Argentina.

An intervention study with a control group was defined. The regions chosen for the program were Anecon Grande, Rio Chico Abajo, Nahuel Pan, Manuel Choique, Blancura Centro and Lipetren. Each farm was defined as an Epidemiologic Unit (EU), each containing one house or houses for one extended family. The geographic region was the Rio Negro Province in Argentina comprising, in total, an area of 5820 Km^2^ [10] (Fig 1).

Among the selected communities there are five health centres, each employing a sanitary agent responsible for the first contact of the centre with the farmers. The centres were located in Manuel Choique (latitude -41.7777 longitude -70.1369), Anecon Grande (-41.3215–70.2742), Rio Chico abajo (-41.7098–70.4761), Nahuel Pan (-41.9004–71.4932) and Blancura Centro (-40.2200–69.4676). In these areas there were originally 192 small farmers; 150 were the subject of the intervention due to their keeping sheep. At the start of the program 16511 sheep were present in the trial regions, ranging from 10 to 200 animals per producer. There were 452 dogs among the 192 EU. In these communities it is common that land is not subdivided with fences, resulting in trans-boundary movement of sheep and dogs.

A baseline for infection in the intermediate and definitive hosts, and also in the environment, was established in 2009 [10], which included serology (ELISA/Western blot) and necropsy in sheep.

The EG95 vaccine used in the study was produced by the University of Melbourne [17]. It was lyophilized and supplied complete (including Quil A adjuvant) in vials containing 50 or 100 doses. The vaccine was rehydrated with sterile distilled water on the morning of the day of use. One ml of reconstituted vaccine containing 50μg EG95 and 1 mg Quil A was injected subcutaneously into the neck of the animal. Vaccine containers with vaccine already diluted were kept in coolers while travelling between the different EUs. Reconstituted vaccine was discarded if not used on the day it was reconstituted. Vaccination activities began in the month of December of 2009.

Separate regions were assigned to one of two different treatments. One group comprising 71 EU in the Blancura Centro and Lipetren regions, was established as a control EU where no vaccinations were undertaken. In the treatment group, comprising 79 EU in the Anecón Grande, Mamuel Choique, Nahuel Pan and Rio Chico abajo regions, lambs received two initial immunizations with EG95 (the first dose was applied to animals at approximately 30 days of age and the second dose at 60 days of age before weaning) and also received a single and final booster immunization at approximately 1–1.5 years of age. Lambs born on these EU in subsequent years underwent the same vaccination treatments.

In order to facilitate the vaccinations, animals were herded into paddocks by the farmers following a visit and instructions from one of the participating community sanitary agents and an announcement was made to the community of the impending visit by the project staff via “National Radio”. All vaccinated animals were tagged with a single ear tag using a different colour tag for each year of the project. Animals were not individually identified; on this basis, an animal with a tag was an animal that received at least one dose of vaccine. Vaccinations were carried out by 4 teams, one of which visited each property twice each year, approximately one month apart. For the duration of the trial, other CE control activities (praziquantel treatment of dogs every 3 months) continued throughout the regions comprising the vaccination areas and control areas, as well as the surrounding regions.

In order to evaluate the effects of the intervention, animals of 6 years old were purchased from farmers in both the intervention and control regions and euthanized. The numbers of animals assessed were determined by their being made available by the owners. The viscera of each animal were carefully inspected for the presence or absence of lesions compatible with *E*. *granulosus* and their anatomic location was registered. The presence of at least one viable CE cyst (containing a cavity with clear fluid and germinal membrane) classified the animal as positive. All the cysts detected at macroscopic examination were separated from the viscera and taken to the National Reference Laboratory for later confirmation in relation to their size and appearance. Calcified and contaminated cysts were collected for histological confirmation

Statistical analyses were performed using the Chi square test between control and vaccinated groups and between pre-intervention animals (2009) and post-intervention (2015) (p value 0.05) unless otherwise specified. Confidence intervals of 95% (CI95%) were estimating using EPIDAT 3.1 (Xunta of Galicia, Spain).

### Ethics Statement

Regarding the ethical treatment of the sheep and dogs used in the study, the research committee of the veterinary school of the National University of La Pampa approved through resolutions 033/210 and 089/2013 the protocol used in each case. The study was conducted adhering to the regulations of the National Animal Health Service concerning animal welfare.

## Results

The numbers of animals involved in the vaccinations during the 6 years in which the program was continuing are shown in Table 1 (total 21447 sheep vaccinates). 9834 lambs received an initial dose of vaccine with a minimum and maximum coverage of 68.2% and 95.9% of the animals respectively, and 55.5 to 100% of the farms; 8082 received the second immunization approximately one month later with a minimum and maximum coverage of 43.3–87.8% of the animals respectively and 68.8–95.0% of the farms. 3531 received the third dose approximately one year later with a minimum and maximum coverage of 71.1 and 96.2% of the animals. To apply the third dose, the initial number of lambs was reduced by approximately 50% in each year (due to the animals being sold, consumed, predated, etc.).

**Table 1:** Number of animals vaccinated with EG95; Rio Negro Province, 2009–2015.

Lamb Cohort	First vaccination (%V^1^ / %EU)	Second vaccination (%V^1^ / %EU)	Third vaccination (%V^1^)	Proportion fully vaccinated %V^2^	Total vaccinations
2009–2010	2725 (86.5/93.7)	2448 (77.8/94.9)	1308 (71.1)	47.8	6481
2010–2011	2138 (68.2/55.5)	1745 (55.5/94.9)	616 (96.2)	36.4	4499
2011–2012	1103 (95.9/100)	498 (43.3/68.8)	348 (92.2)	38.2	1949
2012–2013	1104 (92.4/98.3)	969 (81.0/94.9)	579 (89.4)	66.9	2652
2013–2014	1304 (88.0/98.3)	1017 (68.6/91.7)	680 (93.2)	56.2	3001
2014–2015	1460 (91.3/96.7)	1405 (87.8/95.0)			2865
TOTAL	9834	8082	3531		21447

%V^1^: proportion of the animals available to be vaccinated that were actually vaccinated;

%EU: the proportion of the epidemiological units (EU) that were scheduled to be include in the vaccination programme that actually participated;

%V^2^: proportion of animals receiving the full three-dose vaccination schedule

The prevalence of CE in sheep in the vaccinated area at the start of the trial (2009) was determined by ELISA/Western blot as being 26.2% in 2–4 tooth animals (10) (Table 2). Among 16 old sheep from the vaccinated area (estimated to be >5 years of age in 2009) that were necropsied, 9 were determined to be infected with *E*. *granulosus* (Table 3). In 2011, 7.8% of the 2–4 tooth age class were positive using ELISA/Western blot (p = 0.006, between 2009 and 2011) (Table 2). In relation to the epidemiological units (EU or farmers) in 2009, 80.0% had at least one animal positive to ELISA/Western blot in the 2–4 tooth age class and 42.8% in 2011.

**Table 2:** Diagnostic values for *Echinococcus granulosus* obtained as base-line serology data [10], and impact before and after the introduction of the EG95 vaccine, in sheep and in small holder farms in the Rio Negro control program during 2009/2011.

Animal/technique	Year	Control Area			Vaccination area			Total				P *
		N	Pos	%	N	Pos	%	N	Pos	%	95% CI	
Sheep/2-4 teeth ELISA/WB Individual samples	2009	168	44	26.2	84	22	26.2	252	66	26.2	(2.03 31.3)	1.00
	2011	84	33	39.3	154	12	7.8	238	45	18.9	(13.0 24.1)	<0.0001
P**			0.03			0.0001						
Sheep/2-4 teeth ELISA/WB Farms	2009	9	9	100	25	20	80	34	29	76.3	(68.9 95.0)	0.22
	2011	8	7	87.5	28	12	42.8	36	19	52.8	(35.1 70.5)	0.02
P**		0.47			0.006							

* Chi square test by statistical comparisons between groups control and vaccination

** between groups 2009 and 2011

N: Number of sheep assessed. Pos Number found positive in ELISA or Western blot (WB) to *E*. *granulosus*

**Table 3:** Diagnostic values for *Echinococcus granulosus* obtained as base-line data and impact before and after the introduction of the EG95 vaccine in the Rio Negro control program during 2009/2015.

Animal/technique	Year	Control Area			Vaccination Area			P*
		N	Pos	%	N	Pos	%	95%CI
Sheep/adults Necropsy	2009	46	32	69.6	16	9	56.3	0.57
	2015	32	14	43.7	19	4	21.1	0.1
P**		0.02			0.03			
Cysts / animal	2015	1.5			0.3			0.02***

* Chi square test by statistical comparisons between groups control and vaccination and

** between groups 2009 and 2015;

*** Student’s t-test between control and vaccinated groups

In the vaccination area during 2015, necropsies were performed on 19 sheep, 4 of which (21.1%) were found to have hydatid cysts (Table 3). The difference in prevalence was statistically significant between 2009 and 2015 (p = 0.03). In the control area, out of 32 necropsies conducted in 2015, 13 animals (40.6%) had hydatid cysts; the difference in the prevalence between the two areas being non-significant (p = 0.1).

In the vaccinated animals, 6 hydatid cysts were found in the 4 sheep infected, all of which were small (1 x 1.3 cm to 0.2 x 0.2 cm); 2 were in the liver (one fertile), and 4 in the lung (average 0.3 cysts per animal).

In the control area in 2015, 47 hydatid cysts were found in the 13 infected animals (1.4 cysts per animal, some larger than 5 cm) making the difference in number of cysts between the animals in the control and vaccinated areas statistically significant (p = 0.02). In relation to the EU in the vaccination area in 2009 94.7% had at least one animal positive to necropsy and 23.5% in 2015.

## Discussion

The experience of the pilot vaccination program in Rio Negro with the EG95 vaccine described here found that vaccinated sheep had a significantly decreased prevalence of *E*. *granulosus* infection in adult animals, 21.1% in 2015 compared to 56.3% in 2009 (P = 0.03). A decrease in CE prevalence was also observed in the control area between 2009 and 2015 which was not statistically significant. Limitations in the number of animals available for necropsy may have contributed to there not being a statistically significant difference between the vaccination and control areas in 2015. There was an increased presence of veterinary staff involved in undertaking the trial activities in the control area as well as the vaccination areas over the duration of the study. This is considered to have been a likely to have led to greater compliance of the farmers in treating their dogs with praziquantel and this contributed to the reduction seen in the prevalence of CE there the non vaccination areas. In relation to the number and size of the hydatid cysts, 1.4 cysts per animal were found in the control area in 2009 (in the same communities as Larrieu et al., 2001) whereas 0.3 cysts were found per infected animal in the vaccinated area after 5 years of the program. The number of farmers with at least one *E*. *granulosus* infected animal was 94.7% at the start of the program and 23.5% in the evaluation described here. This suggests a substantial decrease of the infection risk to dogs due to a reduced availability of infected offal, which would be expected to translate to a lower infection in dogs and consequently incidence of human infection in the communities where the vaccine was used.

Evaluation of the EG95 vaccine using experimental infections suggested the potential of the vaccine for reducing *E*. *granulosus* transmission through the parasite’s intermediate hosts [12,13] and that this would decrease the incidence of human infections. Torgerson [14–16] used mathematical models to predict the impact of various options for control of CE and considered that a program involving vaccination of intermediate hosts together with 6-monthly treatment of dogs with praziquantel would decrease the time needed to achieve control of disease transmission. Under field conditions, this is the model that has been applied in this trial in Rio Negro.

In an initial assessment of the pilot vaccination program of Rio Negro with the EG95 vaccine, Larrieu et al. [10] demonstrated that it substantially reduced the prevalence of *E*. *granulosus* infection in animals up to 3 years of age. That initial assessment was made in 275 two year old sheep based on ELISA/WB. Twelve of the 154 vaccinated animals were determined to be positive (7.8%) while in the control area 33 out of 84 sheep were found positive (39.3%). These data cannot unequivocally be inferred to imply infection with *E*. *granulosus* as distinct to only exposure to the parasite. Here we have shown that the level of protection has been sustained, essentially for the lifetime of sheep in the regions involved in the trial. In relation to the prevalence in infection seen on individual farms, in 2009 80.0% had at least one animal positive in the 2–4 tooth age class and in 2011 42.8% of farms had at least one animal positive (p<0.05).

The hydatid cysts that were found in vaccinated animals were small. These small cysts were more typical of recent infections [18], especially in lambs, but in this case were found in adult animals.

One aspect of epidemiological interest was the finding in the vaccination area of one animal that was originally from the control area, not vaccinated, and was found to have multiple *E granulosus* infection at necropsy. It is known that there is relatively frequent exchange of animals amongst farmers. This indicated one source of risk for maintenance of disease transmission in control areas if the territorial coverage of control is not total.

The coverage of vaccination was not 100%. Table 1 summarizes the proportion of the animals that were vaccinated and the proportion of the farms that were able to participate during each instance of vaccination planned. Total numbers of animals available for vaccination at each farm were determined for initial immunizations from the farmers’ knowledge (not all animals eligible for vaccination were made available). This situation was similar in relation to the first, second and third doses of vaccine. The number of animals that were present for vaccination at 1–1.5 years of age was affected by many factors including natural mortality, animals sold/gifted or consumed by the farmers. Compliance for the initial vaccination in the different years of the program ranged from 68.2% to 95.9%. Compliance for the second vaccination ranged between 43.3% and 87.8% and compliance of third vaccination ranged between 71.1% and 96.2%.

Considering the animals from the first year of the program, 86.5% received their first injection, 77.8% received their second vaccination and 71.1% the third vaccination. Hence, among the group of animals that were used to assess the effectiveness of the programme by necropsy, an estimated 47% would have received the intended 3-dose immunization schedule (0.865 x 0.778 x 0.711). It was not possible to determine whether the individual ‘vaccinated’ animals that were assessed at necropsy had actually received all of the three immunizations that were intended. The native communities where this control program was undertaken are remote and have rudimentary infrastructure (10). It was not always possible to communicate with all farmers to ensure compliance at each time vaccinations were due to be undertaken. In many instances it was also not possible for the farmers to have all their animals available for vaccinations when they were due. Animals were not individually identified; they were tagged with a different colour each year, however the presence of a tag on an animal indicated only that it had received at least one vaccination, not necessarily the three immunizations that were intended. This would be expected to adversely affect the level of protection achieved in the sheep in comparison to protection seen in controlled experimental trials [19–21], but represents the realities of working in the field under difficult conditions. Other factors may also have influenced the level of protection that was afforded to the sheep with any vaccination, including poor vaccine application (eg insufficient dose), *E*. *granulosus* infection that may have been acquired by the animals prior to vaccination, undernourished sheep or a decline in the level of protection conferred by the vaccine over time.

These are factors that could hinder the effectiveness of vaccination that are not inherent to the vaccine itself (except decrease of protection). These factors could explain the difference between 90–100% protection in experimental use and 62% (56.3% initial prevalence, 21.1% prevalence after the intervention) in its field use; with existing geographical, social and cultural difficulties in endemic areas.

The resources available to undertake this trial did not allow vaccination of both sheep and goats. Most households in the trial areas kept goats as well as sheep, and goats are a suitable host for *E*. *granulosus*. Control of *E*. *granulosus* transmission in a region such as the one investigated here would likely be more effective if goats were vaccinated as well as sheep, however that would increase the vaccination efforts and costs. In this study, the vaccination model was based in the model with the lowest cost and vaccination effort, with probability of success.

Nevertheless, this trial has demonstrated the EG95 vaccine is a valuable tool to assist with reducing *E*. *granulosus* transmission, even in circumstances where delivery of the program faces many practical difficulties. In the future it will be important to demonstrate the effect of sheep vaccination on transmission to dogs and hence the likely effects on transmission of CE to humans.
